# Humoral Immune Response to SARS-CoV-2 Spike Protein Receptor-Binding Motif Linear Epitopes

**DOI:** 10.3390/vaccines12040342

**Published:** 2024-03-22

**Authors:** Maria E. S. Monteiro, Guilherme C. Lechuga, Paloma Napoleão-Pêgo, João P. R. S. Carvalho, Larissa R. Gomes, Carlos M. Morel, David W. Provance, Salvatore G. De-Simone

**Affiliations:** 1 Center for Technological Development in Health (CDTS), National Institute of Science and Technology for Innovation in Neglected Population Diseases (INCT-IDPN), Oswaldo Cruz Foundation, Rio de Janeiro 21040-900, RJ, Brazil; monteiro.meduarda@gmail.com (M.E.S.M.); gclechuga@gmail.com (G.C.L.); pegopn@gmail.com (P.N.-P.); joaopedrorsc@gmail.com (J.P.R.S.C.); larissagomes@gmail.com (L.R.G.); carlos.morel@fiocruz.br (C.M.M.); bill.provance@fiocruz.br (D.W.P.); 2 Program of Post-Graduation on Parasitic Biology, Oswaldo Cruz Institute, Oswaldo Cruz Foundation, Rio de Janeiro 21040-900, RJ, Brazil; 3 Epidemiology and Molecular Systematics Laboratory (LEMS), Oswaldo Cruz Institute, Oswaldo Cruz Foundation, Rio de Janeiro 21040-900, RJ, Brazil; 4 Program of Post-Graduation on Science and Biotechnology, Department of Molecular and Cellular Biology, Biology Institute, Federal Fluminense University, Niterói 22040-036, RJ, Brazil

**Keywords:** SARS-CoV-2, variants, spike glycoprotein, receptor-binding motif, receptor-binding domain, neutralizing antibodies, Ig subclasses, IgG, IgA

## Abstract

The worldwide spread of SARS-CoV-2 has led to a significant economic and social burden on a global scale. Even though the pandemic has concluded, apprehension remains regarding the emergence of highly transmissible variants capable of evading immunity induced by either vaccination or prior infection. The success of viral penetration is due to the specific amino acid residues of the receptor-binding motif (RBM) involved in viral attachment. This region interacts with the cellular receptor ACE2, triggering a neutralizing antibody (nAb) response. In this study, we evaluated serum immunogenicity from individuals who received either a single dose or a combination of different vaccines against the original SARS-CoV-2 strain and a mutated linear RBM. Despite a modest antibody response to wild-type SARS-CoV-2 RBM, the Omicron variants exhibit four mutations in the RBM (S477N, T478K, E484A, and F486V) that result in even lower antibody titers. The primary immune responses observed were directed toward IgA and IgG. While nAbs typically target the RBD, our investigation has unveiled reduced seroreactivity within the RBD’s crucial subregion, the RBM. This deficiency may have implications for the generation of protective nAbs. An evaluation of S1WT and S2WT RBM peptides binding to nAbs using microscale thermophoresis revealed a higher affinity (35 nM) for the S2WT sequence (GSTPCNGVEGFNCYF), which includes the FNCY patch. Our findings suggest that the linear RBM of SARS-CoV-2 is not an immunodominant region in vaccinated individuals. Comprehending the intricate dynamics of the humoral response, its interplay with viral evolution, and host genetics is crucial for formulating effective vaccination strategies, targeting not only SARS-CoV-2 but also anticipating potential future coronaviruses.

## 1. Introduction

COVID-19 is attributed to severe acute respiratory syndrome coronavirus 2 (SARS-CoV-2), which initially emerged in Wuhan, China, and quickly disseminated globally, leading to substantial economic and societal challenges [1]. Despite the recent announcement by the World Health Organization declaring the end of the COVID-19 pandemic [2], the virus continues to evolve through mutations and recombination. The emergence of new SARS-CoV-2 variants raises concerns regarding their potential to evade host immunity. For instance, the Omicron variants have been reported to escape neutralizing antibodies (nAbs) induced by vaccination or prior infection [3]. Mutations, especially in the Spike (S) protein, threaten the efficacy of current vaccines due to their capacity to evade antibody recognition, a critical component of the immune response [4,5,6]. The study of the humoral response to SARS-CoV-2 is crucial for understanding population-wide and individual responses to viral infection and vaccination. The variation in the antibody response to linear B-cell epitopes among individuals is particularly important for serodiagnosis and vaccine development.

Neutralizing antibodies can target different regions of the S protein, with the majority binding to the N-terminal domain (NTD) and the receptor-binding domain (RBD) [7,8]. The binding modes of receptor-binding domain (RBD)-specific nAbs can be classified into four primary categories, depending on their target epitopes. Antibody neutralization activity can occur through different mechanisms; some of them include competition with the angiotensin-converting enzyme 2 (ACE2) receptor through directly binding to the RBD and the induction of steric hindrance, thereby restraining conformational changes in the Spike protein [9].

The RBD, situated in the S1 domain of the S protein, has undergone extensive examination owing to its marked variability and pivotal function in interacting with the virus-entry receptor in mammalian cells, ACE2. Previous research has shown that the S protein is highly immunogenic, and protective nAb responses are predominantly directed against the RBD [10,11,12].

It is estimated that approximately 90% of the neutralizing activity in convalescent sera is attributed to nAbs binding to the RBD. Within the RBD, the receptor-binding motif (RBM) plays a crucial role in interacting with the ACE2 receptor. The RBM can be divided into three flexible coil regions: knob (aa 444–449 and 496–505), base (aa 490–494 and 450–456), and tip (aa 473–489) [11].

Neutralization is primarily mediated by IgG, IgA, and IgM, with IgG being the most abundant neutralizing antibody [12]. IgM and IgG are produced simultaneously or sequentially in response to SARS-CoV-2 infection, reaching peak concentrations within the first two weeks and remaining in the bloodstream for at least six months [13]. IgG can be subdivided into four subclasses (IgG1, IgG2, IgG3, and IgG4) with diverse effector functions based on the constant region [14]. Viral proteins predominantly elicit IgG1 and IgG3 responses, while IgG2 and IgG4 have been associated with polysaccharide antibacterial responses [14,15]. Regarding binding capacity, convalescent sera IgG3 has been shown to have the most pronounced ability to bind to the SARS-CoV-2 RBD [16]. High levels of anti-RBD IgG4 subclass have been correlated with increased mortality and severe disease [17].

While some SARS-CoV-2 mutations may be neutral or harmful, a subset can enhance viral fitness and enable immune evasion [4]. The S protein and ACE2 interaction is predominantly facilitated by amino acid residues within the RBM, which are the focal points of nAbs [18,19,20]. Contact points of potent nAbs like B38 and CC12.1 overlap with Spike–ACE2 interaction residues [18]. Mutations in the RBM, including E484K and N501Y, whether alone or in combination, have been associated with decreased binding of nAbs [11,20]. Significantly, the N501Y mutation compromises the affinity of neutralizing antibodies while concurrently enhancing the binding affinity to ACE2 [21]. Most nAbs appear to bind to conformational epitopes within the RBD, and antibody responses depend on structurally folded S proteins and the RBD [22]. Protein conformation plays a critical role in immunization as several antibodies that display potent virus-neutralizing activity are generated in response to conformational epitopes [22].

Though numerous research groups have identified nAbs targeting the RBM, more understanding of their impact on the antibody pool in the sera of individuals infected with or vaccinated against SARS-CoV-2 should be understood. However, evidence suggests they are produced in lower frequencies [18,23]. Given the critical role of the RBM in viral entry and ACE2 interaction, it is reasonable to assume that it is highly immunogenic and can provide a structure-based framework for rational vaccine design and the selection of immunotherapeutic agents. Consequently, in this work, RBM sequences were selected for synthesizing and evaluating the humoral response in a panel of vaccinated individuals. To understand the impact of SARS-CoV-2 mutations on host antibody responses, sequences from Omicron BA.4 and BA.5 were also analyzed.

Additionally, the IgG class and subclasses specific to the RBM region were characterized in vaccinated individuals, and the interaction of antibodies with the RBM region was assessed using microscale thermophoresis (MST). A comprehensive peptide analysis of the RBM tip revealed that linear peptides obtained from this region do not induce a strong antibody response (are not immunodominant) for most immunized individuals but are still important in some of them.

## 2. Materials and Methods

### 2.1. Serum Samples

A panel of 31 serum samples were obtained from individuals who received a single dose of the Oxford/AstraZeneca vaccine, and 15 serum samples were collected from individuals who received four doses. Serum from healthy donors, collected before the pandemic, was provided by HEMORIO, a centralized network of blood donor facilities in Rio de Janeiro, Brazil. Serum samples were supplied without identifying information to ensure patient privacy.

### 2.2. Peptide Synthesis

SARS-CoV-2 RBM 15-mer peptides [(S1WT) FERDISTEIYQAGST); S2WT (GSTPCN GVEGFNCYF), and S3WT (YFPLQSYGFQPTNGV)] were chosen for synthesis using the F-moc strategy in a synthesizer machine (MultiPep-1 CEM Corp, Charlotte, NC, USA) [24]. Following sequence assembly, F-moc groups were removed, and the peptide resin (Wang resin) was cleaved and fully deprotected with TFA/H2O/EDT/TIS (94/2.5/2.5/1.0 *v*/*v*, 90 min). The peptide was precipitated with chilled diethyl ether and centrifuged, and the pellet was reconstituted in aqueous AcOH (10% *v*/*v*), dried, and stored as a lyophilized powder. Peptides, when needed, were dissolved in water and centrifuged, and the supernatant was filtered using a centric-10 filter. Single peptides were used without prior purification, with their identity confirmed by MS (MALDI-TOF or electrospray).

### 2.3. Enzyme-Linked Immunosorbent Assay (ELISA)

An in-house ELISA was conducted using Immunolon 4 HB plates (Immunochemistry Technologies, Bloomington, MN, USA). The coating process involved overnight incubation at 4 °C, wherein each well received 500 ng of peptides in a coating buffer (50 mM carbonate–bicarbonate buffer, pH 9.6). After washing with PBS-T (phosphate-buffered saline plus 0.05% Tween 20), the plates were incubated for 1 h at 37 °C with 1% BSA (200 µL) in PBS-T to block free binding sites. Following the dilution of patient serum in PBS-T with 1% BSA (1:25), 100 µL of the diluted serum was applied to immunosorbent plates, which were then incubated at 37 °C for 1 h. Following a series of PBS-T washes, plates were treated with 100 µL of mouse anti-human IgG3 (1:1000) followed by association with antimouse peroxidase (1:15.000) or goat anti-human immunoglobulin classes IgG (1:10.000), IgM (1:10.000), and IgA (1:5000) or subclasses IgG1 (1:1000) and IgG2 (1:1000) associated with the HRP molecule, respectively (Sigma-Aldrich, St Louis, MO, USA). Between each step, the plates were incubated at 37 °C for 1 h. Following the addition of 3,3′,5,5′-tetramethylbenzidine (1-Step™ Ultra TMB-ELISA, Science Biotech Ltd., Lages, SC, Brazil) for 15 min, 0.5 M sulfuric acid was utilized to terminate the reaction. The plate was read 30 min after the Stop Solution was added. The values of empty wells that contained only peptides were deducted from the optical density of the sample.

### 2.4. Purification of RBM Antibodies

Anti-RBM antibodies were purified from a pool (*n* = 10) of sera from individuals who received a single dose of the vaccine through an affinity column (3 cm × 1 cm i.d.) prepared using an RBD SARS-CoV-2 recombinant protein [25] coupled on Sepharose-4B beads, following previously described procedures [26] (Figure S1). Anti-RBM antibodies were eluted with a glycine buffer at a pH of 2.8 and collected in Eppendorf tubes containing a Tris–HCl buffer at a pH of 10, subsequently concentrated on centricon-P30 filters (Sigma Chemical Co., Saint Louis, MO, USA). The protein concentration was determined by measuring absorbance at 280 nm and an extinction coefficient of 13.4. An in-house ELISA was used to analyze the activity of purified antibodies. Immunolon 4 HB plates were coated overnight at 4 °C with 200 ng of multiepitope (Dx-SARS-RBD) recombinant protein containing RBM peptides or non-SARS-CoV-2 sequences (Control). The in-house ELISA followed the earlier methods, but incubation was performed using purified antibodies (100 µg/mL) diluted at 1:1.000.

### 2.5. Microscale Thermophoresis (MST)

Purified nAbs to the RBM regions (Figure S1) were labeled with the fluorescent dye NT-647 using Monolith NT™ Protein Labeling Kits and mixed at a 3:1 molar ratio with an unlabeled protein. For the interaction experiments, the fluorescent anti-RBM antibodies were kept at a constant concentration (12.5 µg/mL), while the concentration of the unlabeled S1WT and S2WT peptides varied from 0.5 µg/mL to 0.12 ng/mL. The assay was performed in PBS containing 0.05% Tween 20, and after a short incubation, the samples were loaded into standard MST NT.115 glass capillaries. *Kd* data were calculated using the NanoTemper software package (MO.Affinity Analysis v2.3) [27].

### 2.6. Protein Thermal Shift Assay

Protein thermal shift assays were performed using a QuantStudio 3 PCR System (Applied Biosystems, Foster City, CA, USA). The experiments used a 0.2 mL MicroAmp^®^ optical eight-strip manufactured by Applied Biosystems. Every step was executed on ice. In each measurement, duplicates or quadruplicates of the samples were analyzed. Each measurement included a negative control free of protein and a positive control obtained with Lysozyme (10 µg) and the peptide S8WT (5 µg). A carbonate–bicarbonate buffer (50 mM, pH 9.6) and a protein thermal shift dye (Applied Biosystems, Foster City, CA, USA) were used in a 4× dye concentration. The plate was sealed with MicroAmp™ Optical Adhesive Film (Applied Biosystems, Foster City, CA, USA) and incubated on ice for 15 min after all components were pipetted into the wells. An Applied Biosystems QuantStudio™ 3 Real-Time PCR System was used, utilizing a gradient rate of 0.05 °C/s and a melt curve filter setting of x2-m2 from 25 °C to 99 °C with continuous data collection. Analyses of the melting curves were conducted with the TSAR application in R version 4.2.1.

### 2.7. Statistical Analysis

Prism software (GraphPad version 6, San Diego, CA, USA) was utilized to analyze the data. Statistical differences were identified using the Kruskal–Wallis test and Dunn’s multiple comparisons tests. Differences were considered significant when the *p*-value was less than 0.05. The reactivity index (RI) was calculated by dividing the absorbance of a given protein by the threshold established by a receiver operation curve (ROC) analysis. A result greater than 1.10 was considered positive, while a result less than 0.90 was considered negative [25]; these are represented by dot lines. Samples with a relative index (RI) value of 1.0 ± 10% were classified as gray-zone samples and deemed inconclusive [25].

## 3. Results

**Figure 1:**
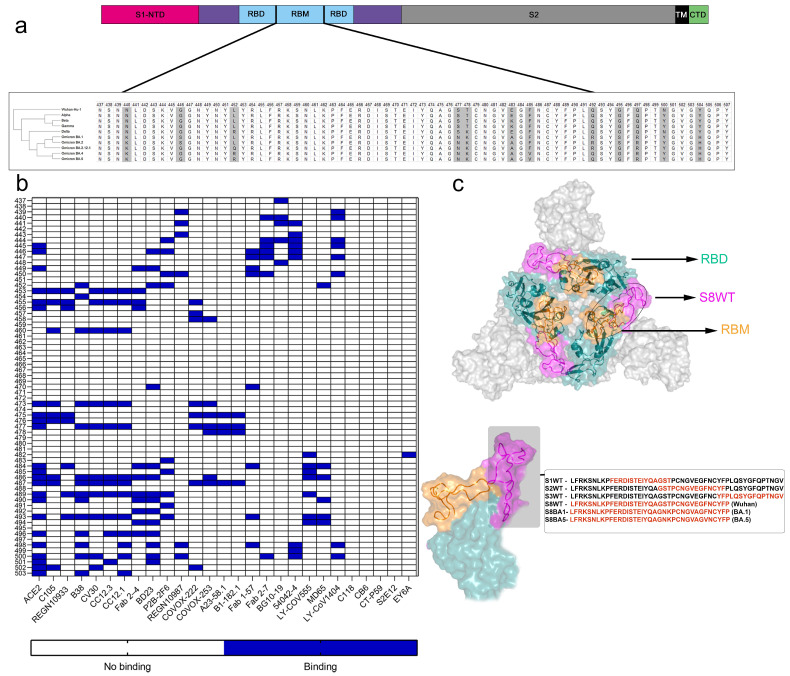
Structural organization of Spike protein and receptor-binding motif (RBM), phylogenetic analysis of VoCs, and interactions of nAbs with residues in RBM. (**a**) Organization of Spike protein domains and phylogenetic analysis of VoCs, highlighting mutations (yellow) in RBM. (**b**) Interactions of RBM residues (437–503) with ACE2 and human nAbs. (**c**) Tridimensional model of S protein trimer shows RBD (green), RBM (yellow), and synthetic peptide S8WT (magenta) comprising RBM tip portion.

The Omicron variants of Concern (VOCs) exhibited unprecedented mutations, especially in the Spike protein. The alignment of the S protein evidenced twelve mutations in the RBM, including mutations previously reported in other VoCs such as L452R, T478K, E484, and N501Y. Additionally, Omicron presented new mutations, namely N440K, G446S, L452Q/R, S477N, T478K, E484A, F486V, Q493R, G496S, and Y505H (Figure 1a), which may be influencing its biological and clinical aspects, such as lower risks of hospitalization and mortality [28], increased transmissibility, reduced replication [29], the protective effects of neutralizing antibodies and enhanced innate immune evasion and suppression [30,31,32]. These last two points also introduce questions about the dynamics of the virus as the convergent evolution of enhanced innate immune antagonist expression as a common pathway of human adaptation. This enhanced expression of immune antagonists is linked to the dominance of Omicron subvariants and their improved innate immune evasion [32].

The interaction of various human-neutralizing antibodies with the binding residues on the RBM was assessed (Figure 1b). Most of the binding occurred on the RBM tip (aa 473–489), where four amino acid residues were mutated in Omicron variants (S477N, T478K, E484A, F486V). Interactions of Spike with ACE2 were highlighted, encompassing residues between 438 and 506 of the RBM, which make maximal contact with the N-terminal peptidase domain (PD) of ACE2. The evaluated human nAbs displayed contact residues in the RBM that overlapped with amino acids interacting with ACE2.

Given the strength of most Omicron mutations in the RBM tip, we synthesized peptides with varying lengths to analyze antibody production and binding affinity. Initially, the RBM, including the tip region, was fragmented into three 15-mer peptides: S1WT (464FERDISTEIYQAGST478), S2WT (476GSTPCNGVEGFNCYF490), and S3WT (489YF PLQSYGFQPTNGV503) (Figure 1c).

To analyze the folding state of the synthesized peptide S8WT, a thermal shift assay (TSA) was performed. Thermal stability was evaluated in a carbonate–bicarbonate buffer at a pH of 9.6. Although peptides can assume many flexible conformations, no transition state was observed (Figure S2), indicating a linear conformation of the peptide S8WT. In contrast, the Lysozyme used as control showed a melting curve with a Tm of 72.7 °C (Figure S2).

**Figure 2:**
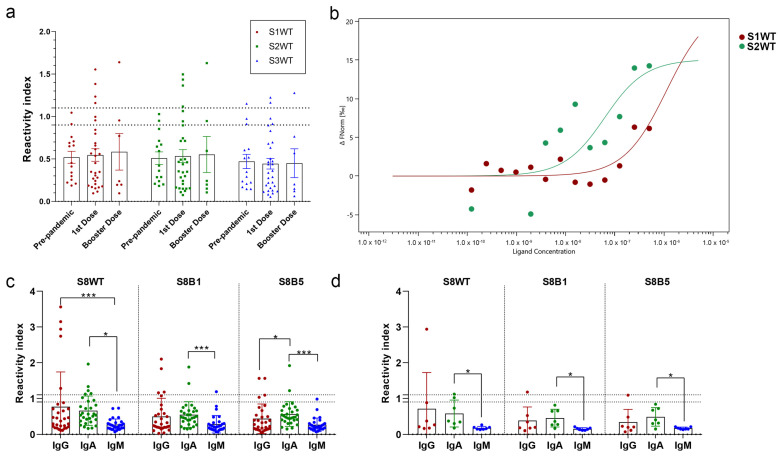
RBM tip humoral response and affinity in vaccinated individuals. (**a**) In-house peptide-ELISAs performed with peptides S1WT, S2WT, and S3WT and a panel of pre-pandemic sera (n = 15), individuals vaccinated with first dose of Oxford–AstraZeneca after 15 days (n = 30) and heterologous booster doses (n = 7). (**b**) Representative of dose–response curve showing binding of antibodies from vaccinated serum with peptides S1WT and S2WT, *Kd* values of 640.7 nM and 35 nM, respectively. Serial dilution of peptide from 0.5 µg/mL to 0.12 ng/mL was used. (**c**) Immunoglobulin class reactivity of RBM peptides S8WT, S8B1, and S8B5, using a panel of vaccinated individuals with the first dose of Oxford–AstraZeneca (n = 30) and (**d**) booster doses (n = 7). Kruskal–Wallis test was applied to identify statistical differences, followed by Dunn’s multiple comparisons tests. *p* < 0.05 was considered a significant difference. * *p* < 0.05 and *** *p* < 0.001.

The seroreactivity of these three regions was tested using a peptide ELISA, using a serum panel of vaccinated individuals with one dose of Oxford/AstraZeneca (ChAdOx1-S) (Table S1) and four doses of heterologous boosting (Oxford/AstraZeneca—ChAdOx1-S; Pfizer-BioNTech—BNT162b2 or Janssen—Ad26.COV2.S) (Figure 2 and Table S2). A panel of pre-pandemic sera was used as a control for the cut-off calculation represented by the dot lines, where a RI > 1.1 represents positive samples for each peptide. In contrast, a reactivity index < 0.9 represents negative samples. Few individuals responded for IgG to the RBM tip peptides, with a reactivity index > 1.1; the seroreactivity values for peptides S1WT and S2WT were similar for prime immunization 4/30 (13.3%) and for heterologous booster doses 1/7 (14.3%). Peptide S3WT demonstrated lower performance, with only two positive samples for first-dose vaccination and one for heterologous booster doses (Figure 2a). Although quantitative antibody detection was low in most vaccinated individuals, one open question is the affinity of produced Spike nAbs to these regions. In this study, microscale thermophoresis was used to measure peptide–antibody interactions’ *Kd*. Purified antibodies specific to the RBM were separated using a multiepitope recombinant protein affinity column (Figure S1), and then the antibodies were tagged with a fluorescent probe; interactions of the antibodies with different concentrations of peptides (S1WT and S2WT) were measured in glass capillaries, and MST traces were recorded (Figure S3). The dissociation constant calculated from the dose–response curve was strikingly different for peptides S1WT (*Kd* = 640.73 nM) and S2WT (*Kd* = 35 nM) (Figure 2b). Results demonstrated that the binding affinity of RBM antibodies to peptide S2WT was stronger. Hence, a 37-mer peptide was synthesized (S8WT, ^452^LFRKSNLKPFERDISTEIYQAGSTPCNGVEG FNCYFP^488^) to increase the sensitivity of the analysis. Additionally, two other peptides from Omicron BA.1 (S8BA1, ^452^LFRKSNLKPFERDISTEIYQAGNKPCNG VAGFNCYFP^488^) and Omicron BA.5 (S8BA5, ^452^LFRKSNLKPFE RDISTEIYQAGNKPCN GVAGVNCYFP^488^) included these VoCs mutations (Figure 1c). Nevertheless, there was a slight increase in seroreactivity for the IgG of the first-dose vaccination 6/30 (20%) for peptide S8WT in comparison with fragmented residues (13–14%) (Figure 2a,c).

Evidently, mutations at the RBM region of Omicron BA.1 (S8BA1) and BA.5 (S8BA5) reduced the reactivity index mean and the number of positive samples (4/30 and 2/30, respectively). IgM levels were low and significantly different from IgG and IgA in single or heterologous doses of vaccinated individuals (Figure 2c,d). Additionally, some individuals with one dose of vaccine produced an IgA response to the RBM tip of the wild-type S (5/30) and a reduced response to Omicron BA.1 (2/30) and BA.5 (2/30) (Figure 2c). In the group of heterologous vaccination booster doses, only one sample had seroreactivity for IgG and IgA against wild-type sequence S8WT (Figure 2d). Mutations in Omicron variants reduced the reactivity index of this sample by almost three times.

The subclasses of IgG-positive samples were also analyzed for the group with the first dose of vaccination; a predominance of IgG1 was found (5/12), but reactivity significantly decreased for Omicron variants (Figure S4).

## 4. Discussion

The humoral response targeting the S protein can be triggered by either natural infection or vaccination, and the efficacy of immune protection against symptomatic SARS-CoV-2 largely depends on the levels of neutralizing antibodies [33]. It has been suggested that nAbs against the S protein might be both limited in quantity and short-lived due to the structural characteristics of coronaviruses [23]. Particularly, the N-terminal and the receptor-binding domain are considered immunodominant and are the primary targets of nAbs. However, nAbs can also recognize regions such as the S2 stem helix (SH) and the S2 fusion peptide [20,34]. Antibodies binding to the RBD contribute to over 90% of the neutralizing activity in convalescent sera [10].

These neutralizing antibodies can be classified into four main classes based on the location of their epitopes within the Spike protein [3]. Although various monoclonal antibodies have been identified to bind to the RBD region [18,35,36,37,38], there still needs to be a greater understanding of the functional diversity of antibodies produced.

The RBM region, comprising the knob, base, and tip, is crucial for ACE2 receptor binding [8]. Many nAbs against SARS-CoV-2 are found to bind to the tip of the RBM, particularly in or around the FNCY patch [21]. Our studies confirm the results of a recent MST analysis on the significance of these residues in antibody binding affinity [25]. Most IgG responses were elicited against a folded RBD, suggesting that conformational epitopes play significant roles in this region and correlate with high levels of virus neutralization.

Recently, a Cryo-EM analysis revealed that a potent neutralizing nAb, CSW1-1805, bound to the loop region (Y473–Y489—RBD ridge) at the RBD ridge, but a single mutation in the Omicron variant, the S477N substitution, reduced the nAb’s activity drastically. The study highlights the importance of the conformations of the RBD and the binding of different nAbs directed to the RBD ridge [39].

Similarly, other works analyzing B-cell epitopes observed that RBM sequences (NGVEGFNCYFP) are less serologically reactive than other S1 and S2 regions [REF—A novel precision-serology assay for SARS-CoV-2 infection based on linear B-cell epitopes of Spike protein]. Analogously to our study, a peptide array revealed that highly immunogenic regions belong to regions outside the RBD [40].

Neutralizing antibodies decline after vaccination and boosting; boosted neutralization titers to Omicron, but not prototypic D614G, decline rapidly [41] and the mechanism behind the decrease and the levels required for individual protection against Omicron and future variants of SARS-CoV-2 are still not clear. Hence, studying different types of nAbs and immunodominant regions of Spike is important for elucidating these open questions and further guiding vaccination strategies [42].

Despite the dominance of conformational epitopes in the RBD [25], our data indicate that immunization also triggers an immune response to linear RBM epitopes in some individuals (~20%) by producing antibodies against them. Linear epitopes to the RBD exhibit a low neutralization profile [25] but possess diagnostic significance, particularly in tests in which proteins may not be correctly folded.

There is concern that variants evade the immune response primarily through mutations in the S protein [43,44,45], compromising both natural and vaccine-induced immunity. For instance, the Omicron variant harbors mutations in the RBM, including ten mutations in this region; some directly interfere with ACE2 binding by affecting crucial residues in the RBM [39,46].

A comparative analysis of the immunogenicity of the RBM, RBD, and the entire S protein reveals reduced seroreactivity in the RBM region [18,23]. However, mouse immunization with RBD or RBM vaccines has shown the induction of neutralizing antibodies [47,48]. According to our findings, adenovirus vaccines containing mRNA, such as AstraZeneca–Oxford or combined heterologous mRNA vaccines, appear to induce low titers of anti-RBM antibodies. This reduced seroreactivity may be attributed to Major Histocompatibility Complex class II (MHC-II) restriction [49].

A study Investigating common MHC-II alleles predicted poor binding for the RBM, suggesting a lack of MHC-II support in T-B cooperation, which impacts the production of nAbs in the general population [48]. Effective antibody responses necessitate cooperation between a B-cell and a CD4 T cell (helper cell) activated by an epitope on the same antigen recognized by the B-cell (T-B cooperation). However, T-B cooperation is constrained by MHC-II molecules [50]. Therefore, studying B- and T-cell epitopes and the HLA polymorphism in diverse populations is crucial for vaccine design [51].

Recent research has demonstrated that the HLA profile influences the variability of both humoral and cellular responses to mRNA vaccines. An association was found between HLA haplotype and a high antibody concentration or low humoral responses to Spike antigens [52]. Another study revealed that variation in the humoral response against SARS-CoV-2 Spike and the RBD 28 days after the first vaccination (ChAdOx1-S) is significantly associated with major MHC-II alleles [53].

## 5. Conclusions

The humoral response, triggered by SARS-CoV-2 infection and vaccination, predominantly targets the Spike protein RBD. However, despite the generation of neutralizing antibodies against Spike, the dominance of the RBD region does not ensure prolonged protective immunity. The emergence of SARS-CoV-2 variants, like the Omicron variant, carrying mutations in the RBD, poses a significant challenge to the effectiveness of existing vaccines. Our findings indicate that the RBM region, which is critical for viral entry and ACE2 interaction, exhibits diminished seroreactivity, potentially affecting the generation of neutralizing antibodies in vaccinated individuals.

The intricate interplay among B- and T-cell epitopes, Major Histocompatibility Complex class II restriction, and Human Leukocyte Antigen polymorphism underscores the necessity for comprehensive population-based studies to inform effective vaccine design. Furthermore, our results reaffirm the importance of RBD conformation in antibody recognition and shed light on the relevance of linear epitopes in a subset of individuals. Understanding the complexities of the humoral response and acknowledging the influence of genetic variability on vaccine efficacy is crucial for devising resilient and inclusive vaccination strategies, as well as diagnostic tests, not only for SARS-CoV-2 but also for emerging coronaviruses.

## 6. Patents

The protein receptacle, method for receptacle production, and antigenic peptide sequences described in this study are protected in Brazil (BR10.2019.017792.6), the USA (PCT/BR2020/050341), Europe (PCT: 26/06/2023), India (PCT: 26/06/2023), and China. (PCT: 26/03/2023) Provisional patents, respectively, were filed by FIOCRUZ. They may serve as a future source of funding.

## Data Availability

The data presented in this study are available on request from the corresponding author.

## Supplementary Materials

The following supporting information can be downloaded at https://www.mdpi.com/article/10.3390/vaccines12040342/s1. Figure S1: Purification of human antibodies against the RBM region of the SARS-CoV-2 spike protein using recombinant RBM Sepharose 4B affinity column (3 × 1 cm, inner diameter). (a) Evaluation of absorbance at 280 nm of different eluate fractions; highlighted in green are eluate fractions used to concentrate polyclonal antibodies. (b) In-house ELISA showing specificity of purified antibodies to multiepitope Dx-SARS-RBD containing RBM fragments. Figure S2: Thermal stability of S8WT peptide. Lysozyme (10 µg) was used as a positive control, and peptide (5 µg) was mixed with Protein Thermal Shift dye. Duplicate and quadruplicate reactions were run on Applied Biosystems QuantStudio™ 3 Real-Time PCR System using melt curve filter setting at x2-m2, continuous data collection, and ramp rate of 0.05 °C/s from 25 °C to 99 °C. Data were analyzed using TSAR application in R software and Thermott (https://thermott.com; accessed on 12 December 2023). Data showed fluorescence vs. temperature (°C). NPC = No protein control. Table S1: One-dose regime; AstraZeneca–Oxford-vaccinated serum information. Table S2: Heterologous booster dose-vaccinated serum information. Figure S3: Microscale thermophoresis (MST) traces of anti-RBM antibodies binding to different concentrations of S1WT (red) and S2WT (green) peptides, determined by microscale thermophoresis (MST). Relative fluorescence (RF) between bound and unbound states was determined over time of 21 s with 20 s MST on time for evaluation. Blue bar indicates ΔRF before temperature gradient was applied; red bar shows ΔRF during thermophoresis. Amount of NT.647-labeled antibodies was kept constant for interaction experiments, while the concentration of unlabeled peptides varied from 0.5 µg/mL to 0.12 ng/mL. Assay was performed in PBS containing 0.05% Tween 20, and after a short incubation period, samples were analyzed in standard glass MST NT.115 capillaries. Figure S4: IgG subclass neutralizing response against RBM peptides in vaccinated sera. Subclass Immunoglobulin subclasses’ reactivity with RBM peptides S1WT, S2WT, S8WT, S8BA1, and S8BA5 using a cohort of vaccinated individuals positive for IgG and with the first dose of Oxford–AstraZeneca (n = 12) and booster doses. For analysis purposes, multiple comparisons were made using Tukey’s multiple comparisons test, where a *p* < 0.05 was considered a significant difference (* = *p* < 0.05).
