# Supplementary material for: Humoral Immune Response to SARS-CoV-2 Spike Protein Receptor-Binding Motif Linear Epitopes
Source: Vaccines (Basel). 2024 Mar 22;12(4):342. doi: 10.3390/vaccines12040342 (PMC11055068; doi:10.3390/vaccines12040342)
Supplement: Supplementary file 1 [file vaccines-12-00342-s001.zip › vaccines-2876875-supplementary.pdf]

– **Supplementary Information** –

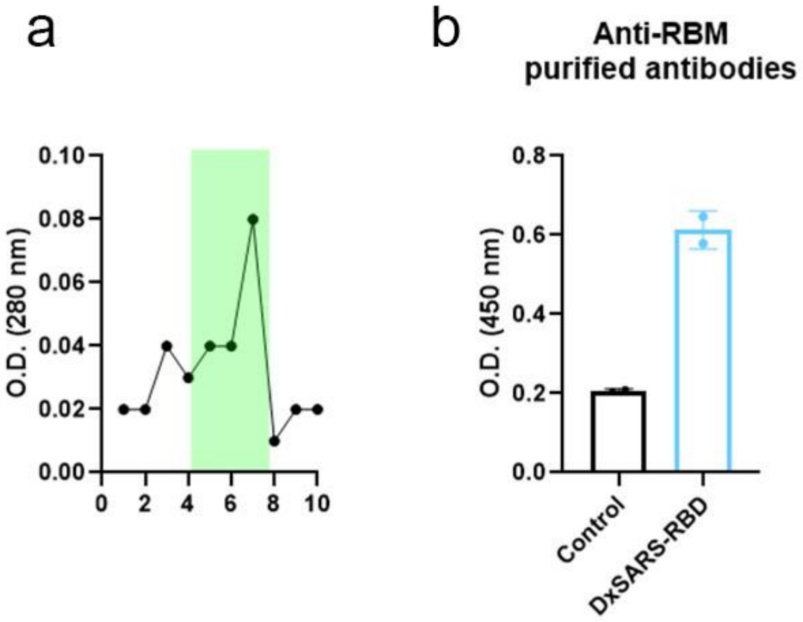

Figure S1: Purification of human antibodies against the RBM region of the SARS-CoV-2 spike protein using a recombinant RBM Sepharose 4B affinity column (3 x 1 cm, inner diameter) (a) Evaluation of absorbance at 280 nm of the eluate different fractions, highlighted in green the eluate fractions used to concentrate polyclonal antibodies (b) In-house ELISA showing the specificity of purified antibodies to the multiepitope Dx-SARS-RBD containing RBM fragments.

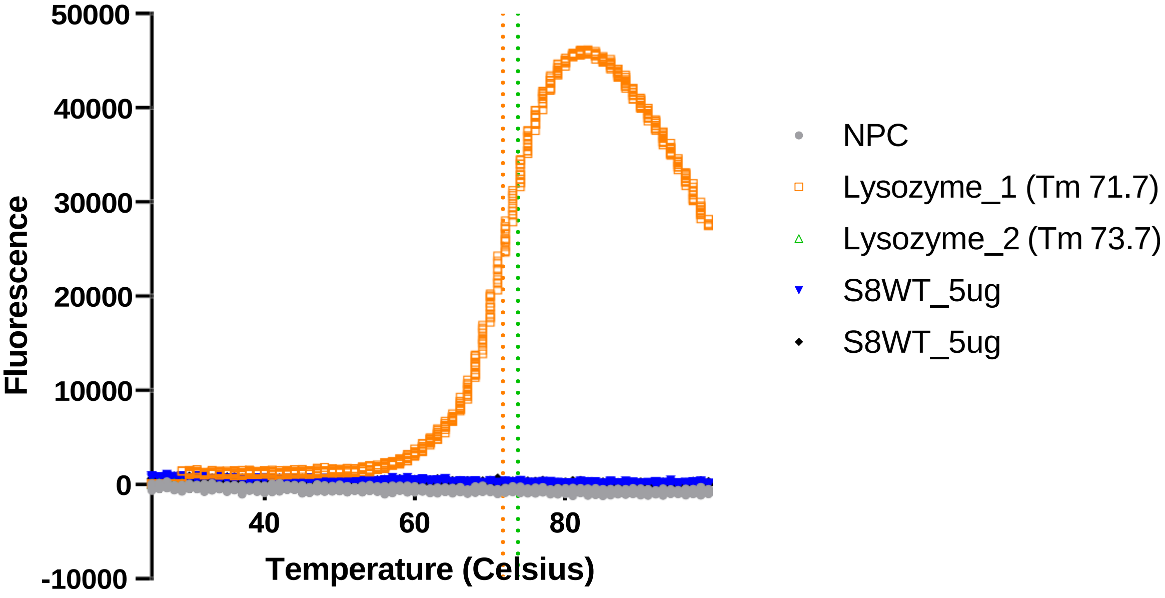

Figure S2: Thermal stability of S8WT peptide. Lysozyme (10 ug) was used as positive control, and peptide (5 ug) was mixed with the Protein Thermal Shift dye. Duplicate and quadruplicate reactions were run on an Applied Biosystems QuantStudio™ 3 Real-Time PCR System using melt curve filter setting at x2-m2, continuous data collection, and a ramp rate of 0.05°C/sec from 25°C through 99°C. Data were analyzed using the TSAR application in R software and Thermott (https://thermott.com). Data showed Fluorescence vs. temperature (°C). NPC = No protein control.

Table S1: One-dose regime AstraZeneca-Oxford vaccinated serum information.

Sample	Gender	1st Dose	Collected	Days after vaccination
**M1**	F	2021-06-18	2021-09-26	100
**M3**	F	2021-07-20	2021-09-26	70
**M4**	F	2021-08-07	2021-09-26	50
**M5**	M	2021-06-21	2021-09-26	97
**M7**	F	2021-09-09	2021-09-26	17
**M9**	M	2021-09-02	2021-09-26	24
**M10**	M	2021-09-15	2021-09-26	11
**M11**	M	2021-08-11	2021-09-26	46
**M12**	F	2021-06-26	2021-09-26	92
**M13**	M	2021-06-27	2021-09-26	91
**M14**	F	2021-06-28	2021-09-26	90
**M17**	M	2021-06-29	2021-09-26	89
**M20**	M	2021-06-30	2021-09-26	88
**M21**	M	2021-07-01	2021-09-26	87
**M23**	M	2021-07-28	2021-09-26	60
**M24**	F	2021-08-03	2021-09-26	54
**M25**	F	2021-07-04	2021-09-26	84
**M27**	F	2021-08-18	2021-09-26	39
**M28**	F	2021-08-20	2021-09-26	37
**M29**	F	2021-09-09	2021-09-26	17
**M30**	F	2021-07-08	2021-09-26	80
**M31**	F	2021-07-16	2021-09-26	72
**M34**	F	2021-08-17	2021-09-26	40
**M37**	F	2021-08-20	2021-09-26	37
**M39**	F	2021-09-10	2021-09-26	16
**M40**	F	2021-08-30	2021-09-26	27
**M41**	F	2021-08-13	2021-09-26	44
**M43**	F	2021-08-22	2021-09-26	35
**M46**	F	2021-08-24	2021-09-26	33
**M47**	F	2021-07-17	2021-09-26	71
**M48**	M	2021-09-12	2021-09-26	14

Table S2: Heterologous booster dose vaccinated serum information.

Sample	Age (years)	Gender	Dose regimen	Last dose	Collected	Days after the last vaccine
B1	39	M	A/A/A/P	2022-07-15	2023-03-08	236
B2	31	F	A/A/P/P	2022-06-22	2023-03-08	259
B3	32	M	A/A/P/A	2022-10-17	2023-03-08	142
B4	26	M	A/A/P/P	2022-11-16	2023-03-08	122
B5	42	F	A/A/P/J	2022-06-09	2023-03-08	303
B6	45	F	A/A/P	2022-01-10	2023-03-08	401
B7	30	F	A/A/P/A	2022-06-20	2023-03-08	261

*A= Astrazeneca viral vector vaccine; P = Pfizer Spike mRNA vaccine; J = Janssen virus-based technology vaccine

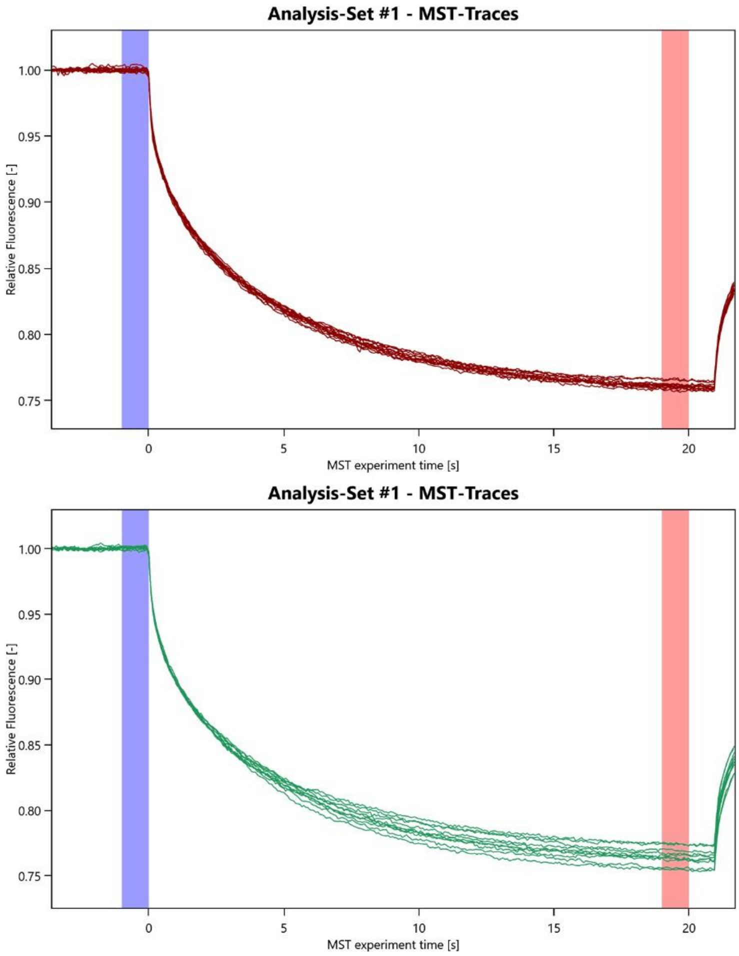

Figure S3: Microscale thermophoresis (MST) traces of anti-RBM antibodies binding to different concentrations of S1WT (red) and S2WT (green) peptides by MicroScale Thermophoresis (MST). Relative Fluorescence (RF) between the bound and unbound states was determined over 21 seconds with 20 seconds of MST-on time for evaluation. The blue bar indicates the ΔRF before the temperature gradient was applied, whereas the red bar shows the ΔRF during the thermophoresis. The amount of NT.647-labeled antibodies was kept constant for interaction experiments, while the concentration of unlabeled peptides varied from 0.5 µg/mL to 0.12 ng/mL. The assay was performed in PBS containing 0.05% Tween 20, and after a short incubation period, the samples were analyzed in

standard glass MST NT.115 capillaries.

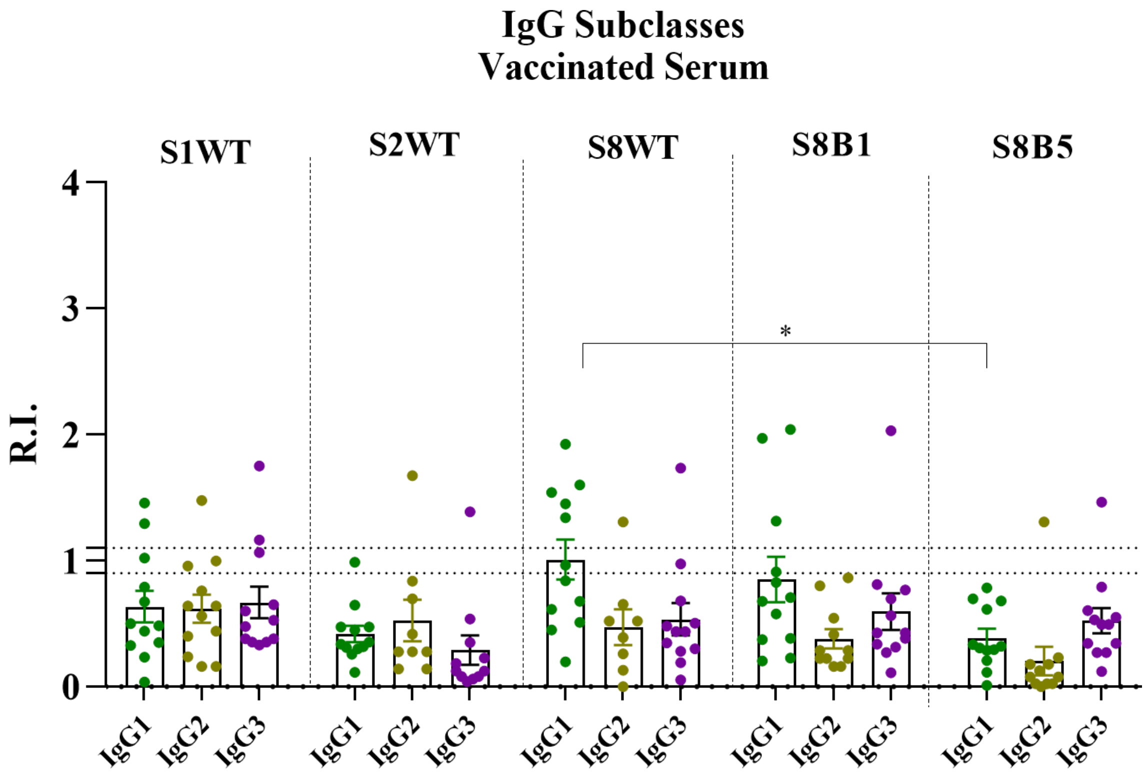

Figure S4: IgG subclass neutralizing response against RBM peptides in vaccinated sera. Subclass Immunoglobulin subclasses reactivity of RBM peptides S1WT, S2WT, S8WT, S8BA1, and S8BA5 using a cohort of vaccinated individuals positives for IgG and with the first dose of Oxford-AstraZeneca (n=12) and booster doses. For analysis purposes, multiple comparisons were made using Tukey's multiple comparisons test, where a $p < 0.05$ was considered a significant difference (* = $p < 0.05$).
